# High frequency of mitochondrial genome instability in human endometrial carcinomas

**DOI:** 10.1038/sj.bjc.6601110

**Published:** 2003-08-12

**Authors:** V W S Liu, H J Yang, Y Wang, P C K Tsang, A N Y Cheung, P M Chiu, T Y Ng, L C Wong, P Nagley, H Y S Ngan

**Affiliations:** 1 Department of Obstetrics and Gynaecology, Queen Mary Hospital, University of Hong Kong; 2 Department of Pathology, Queen Mary Hospital, University of Hong Kong, Pokfulam Road, Hong Kong; 3 Department of Biochemistry and Molecular Biology, Monash University, Clayton, Victoria 3800, Austraila

**Keywords:** mitochondrial DNA, D-loop, mitochondrial microsatellite instability, endometrial carcinoma

## Abstract

To investigate the occurrence of somatic mitochondrial DNA (mtDNA) mutations in human primary endometrial carcinomas, we sequenced the D-loop region, the 12S and 16S rRNA genes of mtDNA of cancer tissues and their matched normal controls. About 56% (28 out of 50) of cases carry one or more somatic changes in mtDNA including deletion, point mutation and mitochondrial microsatellite instability (mtMSI), namely the change in length of short base-repetitive sequences of mtDNA. In particular, mtMSI was frequently detected in 89% (25 out of 28) of all the cases carrying somatic changes followed by point mutations (25%; seven out of 28) and deletion (3.5%; one out of 28). The CCCCCTCCCC sequences located in the Hypervariable Regions I and II of the D-loop and 12S rRNA gene are instability hot spot regions in endometrial carcinomas. It is suggested that errors in replication may account for the high frequency of mtMSI in human endometrial carcinomas. The relatively high prevalence of mtMSI may be a potential new tool for detection of endometrial cancer.

The occurrence of somatic mitochondrial DNA (mtDNA) mutations in various human cancers has recently been reported (Polyak *et al*, 1998; Fliss *et al*, 2000; Jeronimo *et al*, 2001; Jones *et al*, 2001; Kirches *et al*, 2001; Liu *et al*, 2001; Maximo *et al*, 2001; Parrella *et al*, 2001). The genetic changes include deletions, point mutations and mitochondrial microsatellite instability (mtMSI), defined as the change in length in short base-repetitive sequences of mtDNA. Most of the mtDNA mutations were point mutations involving T → C or G → A transitions, probably induced by oxidative stress. In contrast, DNA deletions and mtMSI, which might be generated during erroneous replication, occurred sporadically.

This D-loop region is often selected for study in view of its tendency to accumulate mutations more rapidly than other regions of mtDNA. In our previous study, we sequenced the D-loop region (nucleotide position (np) 16024–576) of 15 pairs of ovarian tumour and matched normal samples and found a total of nine somatic mutations in three samples (20%; three out of 15) (Liu *et al*, 2001). In addition, in the same study, we completely sequenced the mitochondrial genomes of 10 ovarian tumour samples and found six somatic mutations, respectively, in six samples (60%; six out of 10) (Liu *et al*, 2001). Four point mutations and one mtMSI out of the six mutations were detected in the region containing the D-loop, 12S and 16S rRNA genes. Indeed, consistent with other studies, the majority of the mtDNA mutations have been found in the D-loop and the adjacent genes in several tumours (Fliss *et al*, 2000).

So far, the role of mtDNA in endometrial carcinomas has not been reported. To further our investigation on the occurrence of mtDNA mutations in human cancers, we selectively (based on our previous results) sequenced the D-loop, the 12S and 16S rRNA genes (spanning from np 16024–3229) of mtDNA of endometrial carcinomas.

## MATERIALS AND METHODS

Fifty frozen samples of primary endometrial carcinomas and those of their matched normal tissues (including cervix and/or lymphocytes) obtained after surgery were retrieved without specific selection from our tissue bank and used for DNA analysis in this study (approved (No.: EC 1517-00) by the Ethics Committee of the University of Hong Kong). In addition, pathologic paraffin-embedded tumour tissues and sera (if available) from the same set of individuals were also used to cross-check the DNA changes found. DNA was extracted from frozen tissues, paraffin-embedded tumour tissues and sera by the methods described previously (Liu *et al*, 2001). The segment of mtDNA encompassing the D-loop, 12S and 16S rRNA genes was amplified from tissue extracts by long-range and high-fidelity PCR amplification system under conditions as recommended by the manufacturer (Roche Diagnostics GmbH, Mannheim, Germany). Sequencing of the PCR products was carried out using the same procedure as we used in our previous study of mtDNA mutations in ovarian carcinomas (Liu *et al*, 2001). DNA sequences were analysed using Dnasis software. In addition, electropherograms were read manually to identify mutant sequence present at a low level. From our experience, an mtDNA sequence constituting as low as 15% in a heteroplasmic sample (containing a mixture of mtDNA sequences) can be detectable by DNA sequencing. To confirm the DNA changes found in frozen tissues, the corresponding mtDNA fragments within DNA extracted from paraffin-embedded tissues and serum from the corresponding individuals were also analysed. Although paraffin-embedded DNA may undergo some degradation after chemical treatment for fixation, the high copy number of mitochondrial genomes per cell makes PCR amplification of short mtDNA fragments readily achievable, yielding reproducible data. Details of PCR primers and sequencing primers are available upon request from the authors.

## RESULTS AND DISCUSSION

**Figure 1:**
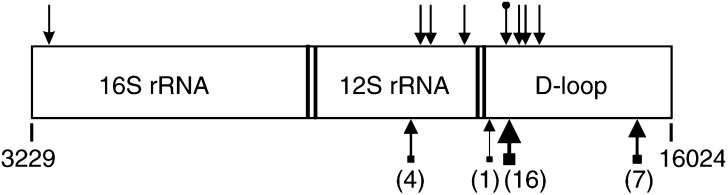
Location of somatic mtDNA mutations detected in a particular region (np from 16024–3229) of mitochondrial genome in endometrial carcinomas. The locations of the mtDNA mutations are indicated by arrows (positions not drawn exactly to scale). The arrows on top indicate the positions of the seven point mutations and the 50-bp deletion (arrow with a black circle at the tail). The arrows at the bottom indicate the positions of the mtMSI. Numbers in parentheses indicate the number of mtMSI detected out of 50 endometrial tumours. Gaps between genes indicate the positions of tRNAs.

Fifty samples of primary endometrial carcinomas and their matched normal controls were studied to determine whether mtDNA mutations could be identified. Indeed, 56% (28 out of 50) of the endometrial tumours were found to carry one or more somatic mtDNA mutations including deletions, point mutations and mtMSI (Figure 1

; Table 1

**Table 1:** Somatic mtDNA mutations found in human endometrial carcinomas

**Code no.**	**Gene**	**Location**	**Mutation type**	**Wild type^a^**	**Normal**	**Tumour**
U9	D-loop	289–346	Deletion	Normal length	Normal length	50-bp deletion
G12	D-loop	152	Point mutation	T	T	C
U34	D-loop	251	Point mutation	G	G	G, A
U10	D-loop	294	Point mutation	T	T	T, C
U27	12S RNA	650	Point mutation	T	T	T, C
U59	12S RNA	817	Point mutation	G	G	A
G29	12S RNA	879	Point mutation	T	T	C
U44	16S RNA	3163	Point mutation	G	G	A
U111	D-loop	514–523	mtMSI	(CA)_5_	(CA)_4_	(CA)_5_
U24	12S RNA	956–960	mtMSI	C5	C5	C4, C5
U32	12S RNA	956–960	mtMSI	C5	C6	C6, **C7**
U120	12S RNA	956–960	mtMSI	C5	C6	C7
U27	12S RNA	956–965	mtMSI	CCCCCTCCCC	**C11**, C12, C13, C14	**C12**, C13, C14
U10	D-loop	303–309	mtMSI	C7	**C8**, **C9**	C8
U12	D-loop	303–309	mtMSI	C7	C7, **C8**, **C9**	C8
U24	D-loop	303–309	mtMSI	C7	**C8**, **C9**	**C9**, **C10**
U42	D-loop	303–309	mtMSI	C7	C7, **C8**, C9	**C8**, **C9**, C10
U44	D-loop	303–309	mtMSI	C7	C7, **C8**, C9	C8, C9, **C10**, C11
U46	D-loop	303–309	mtMSI	C7	C7, **C8**, C9	**C8**, **C9**, **C10**, C11
U52	D-loop	303–309	mtMSI	C7	C7, **C8**, C9	**C8**, **C9**, C10
U55	D-loop	303–309	mtMSI	C7	C8, **C9**, C10	**C9**, **C10**
U56	D-loop	303–309	mtMSI	C7	**C8**, **C9**	C8
U109	D-loop	303–309	mtMSI	C7	**C8**, **C9**, **C10**, C11	**C8**, C9
U120	D-loop	303–309	mtMSI	C7	**C8**, **C9**, C10	C8
U125	D-loop	303–309	mtMSI	C7	**C8**, C9, C10	**C8**, C9
G24	D-loop	303–309	mtMSI	C7	**C7**, C8, C9	**C7**, **C8**
G27	D-loop	303–309	mtMSI	C7	C7	C7, **C8**
G35	D-loop	303–309	mtMSI	C7	**C9**, C10	C9, **C10**, C11
G81	D-loop	303–309	mtMSI	C7	C8, **C9**, C10	C9, **C10**, C11
U9	D-loop	16184–16193	mtMSI	CCCCCTCCCC	C13	C14
U25	D-loop	16184–16193	mtMSI	CCCCCTCCCC	C11	C12
U37	D-loop	16184–16193	mtMSI	CCCCCTCCCC	C12	C10, C11, C12
U44	D-loop	16184–16193	mtMSI	CCCCCTCCCC	C10	C10, **C11**, **C12**
U51	D-loop	16184–16193	mtMSI	CCCCCTCCCC	C11, **C12**, C13	C12, **C13**, C14
U58	D-loop	16184–16193	mtMSI	CCCCCTCCCC	C12	C11
G29	D-loop	16184–16193	mtMSI	CCCCCTCCCC	C7	C7, **C8**, C9, C10

Table entries in bold font indicate the predominant sequence variants in heteroplasmic mixtures.

a Wild type indicates the nucleotide(s) in the Revised Cambridge reference sequence (GenBank NC_001807).

). Somatic mtDNA mutations are defined as those present in the tumour but not in the normal tissue from the patient concerned. The revised Cambridge sequence (wild type) of the relevant position, in each case, is shown for reference in Table 1. The mtDNA in normal tissue of individual patients constitutes the mitochondrial haplotype but may differ from this reference sequence, thereby representing a germline polymorphism.

In just one (U9; Table 1) of the 50 tumour samples, a homoplasmic mtDNA deletion involving a pair of 9-bp direct repeats (CCAAACCCC) located at np 298–306 and np 348–356 was detected in the D-loop. The mtDNA mutation deleted 50-bp of DNA sequence including the Conserved Sequence Block (CSB) II starting at np 298 with one of the direct repeats retained.

In addition, a total of seven somatic point mutations (14%; seven out of 50) were detected in the current set of endometrial tumour samples (Table 1). Three different mutations were detected in the D-loop, one in each of the samples G12, U34 and U10. Another three mutations were detected in the 12S rRNA gene (in U27, U59 and G29) and a further one in the 16S rRNA gene (in U44). They were all novel mtDNA mutations except the one at np 152 of G12 (Liu *et al*, 2001). The mutations were all either T → C or G → A transitions.

Other than deletion and point mutations, a high frequency (50%; 25 out of 50) of mtMSI was detected, especially in the D-loop region in these 50 endometrial tumour samples. We observed mtMSI in four different regions (Table 1), including CA repeats at np 514–523 within a nonfunctional D-loop sequence and a common homopolymeric C stretch interrupted by a T (CCCCCTCCCC) at each of the following locations: np 956–965 within the 12S rRNA gene; np 303–315 within the CSB II; and np 16184–16193 at the 3′ end of the termination-associated sequence.

**Figure 2:**
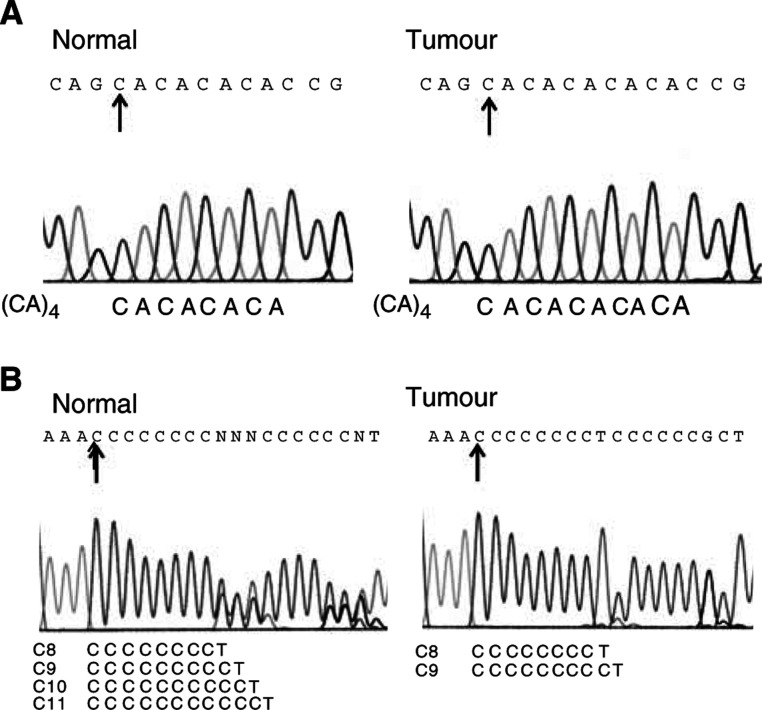
Detection of mtMSI in endometrial carcinomas by DNA sequencing. (**A**) The normal sequence (commencing at np 514, arrows) of U111 (Table 1) is a microsatellite DNA containing four CA repeats. In the tumour, it was expanded to carry five CA repeats. (**B**) The mtDNA sequence found in the normal tissue of U109 (Table 1) contained heteroplasmic stretch of C residues, comprising C8, C9, C10 and C11 (commencing at np 303, arrows). In the tumour, mtDNA sequence contained mainly C8 and a small proportion of C9.

The sequencing details illustrating two different features of the occurrence of mtMSI are depicted in Figure 2

. The normal DNA of sample U111 (Figure 2A) is homoplasmic containing a consecutive set of four CA dinucleotides starting from np 514. In the tumour, the sequence has expanded to five CA dinucleotides. This CA-repeat instability was only found in U111 (2%; one out of 50) (Table 1). A more complex example of mtMSI is shown in Figure 2B. The normal DNA of sample U109 is heteroplasmic. It contains stretches of cytosine residues of different lengths starting from np 303 (C8, C9 and C10 are in similar proportions but C11 is a minority species). In the tumour, the relative predominance of these length variants has shifted markedly, the mtDNA sequence now containing mainly C8 and a small proportion of C9. The use of DNA sequencing to detect mtMSI allows us to determine exactly the somatic changes and to estimate the relative proportion of each type of sequence within a heteroplasmic sample.

Novel mtMSI located within the 12S rRNA gene (Table 1) was found in the present study. The changes were either the deletion/insertion of one cytosine residue at the homopolymorphic C tract in front of the thymine (detected in U24; U32; U120) or the germline T → C polymorphism producing an extra long homopolymorphic C tract leading to DNA instability (detected in U27). The frequency of occurrence was 8% (four out of 50).

We observed mtMSI in two other regions in the D-loop. Changes in length of the homopolymorphic cytosine at np 303–309 were detected in 32% (16 out of 50) of endometrial carcinoma samples. Another mtMSI involves the CCCCCTCCCC sequence at np 16184–16193 at the 3′ end of the termination-associated sequence and within the 7S DNA binding site. The germline T → C polymorphism at np 16 189 produced an uninterrupted C tract that was unstable in the tumour (Table 1). The frequency of occurrence was 14% (seven out of 50).

The mtMSI within the mononucleotide repeat at np 303–309 is frequently observed in endometrial tumour samples (32%; 16 out of 50). This mtMSI was also detected in ovarian carcinomas (Liu *et al*, 2001) and other human tumours (Sanchez-Cespedes *et al*, 2001) with an average frequency of 22% (55 out of 247). In concordance with the data of Sanchez-Cespedes *et al* (2001), patients carrying mtDNA instability at this particular region had higher levels of heteroplasmic length variants in the normal tissues than patients not showing instability. Heteroplasmic length variants of this region were found to be quite constant during life (Lagerstrom-Fermer *et al*, 2001). This region is part of the CSB II and the binding site of replication primer. It is not clear if the relatively unstable somatic changes would alter mtDNA replication in the tumour cell.

The germline T → C polymorphism at np 16189 producing an uninterrupted C tract was unstable in the endometrial tumour. The frequency of occurrence was 14% (seven out of 50). This particular mtMSI has been reported in glioblastoma (Kirches *et al*, 2001) at a frequency of 23% (four out of 17). These sequence variants when in the mitochondrial haplotype have been considered as a significant genetic predisposing factor for Type II diabetes (Poulton, 1998). Indeed, we have recently observed (Liu *et al*, 2003) an elevated frequency of this haplotypic variant in patients with endometrial carcinomas, suggesting that this may also be a possible genetic predisposing factor for such human cancers.

The data presented here indicate a remarkably high frequency of somatic mtDNA mutations in endometrial carcinomas. Although the entire mitochondrial genome was not sequenced, this particular stretch of mtDNA may be a hot spot region of somatic mutations. The most abundant type of mutation observed in the D-loop region of endometrial carcinomas is mtMSI. While the mtDNA sequence variants representing the somatic mutations occurring here, including point mutations and mtMSI variants, are often found as germline polymorphisms in the human population, it is worthwhile considering relevant issues including their possible differential occurrence in various cancers, their proposed mechanisms of formation and their potential clinical significance.

Variation in the CA repeats was detected in only one (2%; one out of 50) of the endometrial carcinoma samples. Instability of the CA repeats was also found at a low frequency in ovarian (8%; two out of 25) (Liu *et al*, 2001), gastric (19%; six out of 32) (Maximo *et al*, 2001) and glioblastoma (6%; one out of 17) (Kirches *et al*, 2001) cancers. However, there is a significantly higher frequency of the CA-repeat instability in breast cancer (42.5%; 17 out of 40) (Richard *et al*, 2000). On the basis of these findings, we suggest that somatic mtMSI occurs differentially in different tumours.

Malfunctions of mismatch repair genes have been associated with nuclear MSI. However, to date, no mismatch repair genes have been found responsible for the maintenance of mammalian mitochondrial genome. The occurrence of high frequency (50%; 25 out of 50) of mtMSI in endometrial tumours indicates that DNA repair in mitochondria is highly inefficient after malignant transformation. The different frequencies of occurrence of the mtMSI at the mononucleotide repeat at np 303–309 in different tumour cell types suggests that deficits in mismatch repair function in mitochondria may vary from one tumour to another.

We detected a higher frequency of mtMSI than point mutations in human endometrial carcinomas. This may be explained by the different mechanisms of mtDNA alteration and/or repair in different tissues during carcinogenesis. The occurrence of mtMSI may derive mainly from erroneous replication, whereas point mutations may be attributed to oxidative damage caused by reactive oxygen species. In addition, a recent study (Longley *et al*, 2001) suggests that the homopolymorphic nucleotide tracts in mtDNA are error-prone because of the low frameshift fidelity of the DNA polymerase gamma that carries out the mtDNA replication. Thus, mtMSI in endometrial carcinomas may be mainly attributed to errors of replication. Moreover, there is supportive evidence that there are cell-specific differences in the repair of mtDNA damage (LeDoux and Wilson, 2001). The differential occurrence of mtDNA mutations in various cancers may be partly due to the prominence of different repair mechanisms in different tumour cell types.

The active generation of new mtDNA variants by replication error and inefficient repair is not the only explanation for the appearance of mtMSI. Somatic mtDNA mutations accumulated in tumours have been proposed to be the result of random genetic drift (Coller *et al*, 2001; Jones *et al*, 2001). This means that the pre-existing sequence variants, which could be present at a low level among the heteroplasmic population of mtDNA in the progenitor cells, could have outgrown, without any selection, other mtDNA molecules after the hundreds or thousands of cell division cycles leading to the clinically manifested tumour. Since many somatic mutations were found in mtDNA sequences without functional significance, for example, within the D-loop region, the presence of somatic mtDNA mutations in tumours might not necessarily lead to mitochondrial dysfunction. In this view, random genetic drift could satisfactorily explain the occurrence of somatic mtDNA mutations during carcinogenesis. A more detailed treatment of these ideas has recently been presented by Chinnery *et al* (2002).

Nevertheless, it is still open to question whether there might be a functional significance of the various mtMSI, point mutations and deletions that are found in cancer tissues as homoplasmic somatic mutations. It is conceivable that some mutations may be functionally selected during neoplastic expansion. The possible functional impact of somatic mtDNA mutations in cancer should therefore be investigated, especially concerning those mutations lying outside noncoding or nonconserved regions.

**Figure 3:**
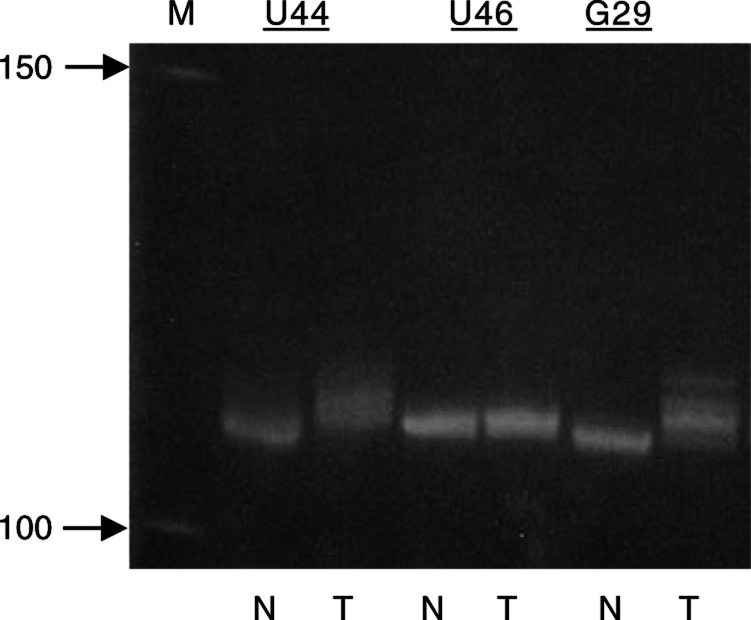
Detection of mtMSI in endometrial carcinomas by polyacrylamide gel electrophoresis. A homopolymeric C stretch interrupted by a T (CCCCCTCCCC) at 16184–16193 was analysed. As a control, U46 carried the wild-type sequence in this segment of mtDNA in both the normal (N) and tumour (T) tissues. The PCR product is a single band of 109 bp and band shifting was not observed. In contrast, both U44 and G29 carried a germline polymorphism T to C at np 16189 to produce an interrupted C stretch. Instability at this C stretch gave rise to heteroplasmic sequences and resulted in band shifting or smearing in PCR products derived from tumour samples. In sample U44, PCR product raised from normal tissue was a single band carrying C10, while the multiple PCR products (carrying C10, C11, and C12) were seen in the tumour sample, giving rise to a smeared band on the gel. Similarly, in sample G29, PCR product raised from normal was a single band carrying C7, while multiple PCR products (carrying C7, C8, C9 and C10) were seen in the tumour sample. The lane marked (M) contains 50 bp DNA markers (sizes of two bands shown in bp).

Finally, in a clinical context, the high frequency of mitochondrial genome instability, in combination with PCR-based assays of high sensitivity, may be a potential new tool for endometrial cancer detection (Parrella *et al*, 2001). Somatic mtMSI involving the change in DNA length, even though the difference is only 1 or 2 bp, can be readily detected by polyacrylamide gel electrophoresis (Figure 3

). In general, the bandshifts or band spreading observed by gel electrophoresis after PCR amplification of tumour mtDNA reflects the distribution of novel or multiple sequence variants in the tumour compared to normal tissue from the same patient (see legend to Figure 3 and relevant parts of Table 1). One particular mutation, the mtMSI variant at the mononucleotide repeat at np 303–309 (Table l; see also foregoing discussion), because of its frequent occurrence in various cancers, may be considered as a potential new tool for early cancer detection. In this case, the application of PCR coupled with gel electrophoresis lends itself to rapid analysis of multiple samples.
